# Ubiquitination‐Associated Ductal–Fibroblast Crosstalk Shapes Tumor Progression and Prognosis in Pancreatic Ductal Adenocarcinoma

**DOI:** 10.1155/humu/1665552

**Published:** 2026-04-30

**Authors:** Yiping Fu, Xin Zhou, Lai Jiang, Shengke Zhang, Yuheng Gu, Ziye Zhuang, Gang Huang, Zhulin Xu, Pei Xu, Xiaolin Zhong

**Affiliations:** ^1^ Clinical Medical College, Southwest Medical University, Luzhou, China, swmu.edu.cn; ^2^ Clinical Skills Center, The Affiliated Hospital of Southwest Medical University, Luzhou, China, ahswmu.cn; ^3^ Department of General Surgery, West China Hospital, Sichuan University, Chengdu, China, scu.edu.cn; ^4^ Liver Transplant Center, Transplant Center, West China Hospital, Sichuan University, Chengdu, China, scu.edu.cn; ^5^ First Clinical Medical College, Guangdong Medical University, Zhanjiang, Guangdong, China, gdmu.edu.cn; ^6^ Department of General Surgery (Hepatopancreatobiliary surgery), The Affiliated Hospital of Southwest Medical University, Luzhou, China, ahswmu.cn; ^7^ Department of Pediatric Surgery, The Affiliated Hospital of Southwest Medical University, Luzhou, China, ahswmu.cn; ^8^ Department of Gastroenterology, Affiliated Hospital, Southwest Medical University, Luzhou, China, swmu.edu.cn

**Keywords:** disease prognosis, ductal-fibroblast crosstalk, multiomics, pancreatic ductal adenocarcinoma (PDAC), ubiquitination

## Abstract

**Background:**

Pancreatic ductal adenocarcinoma (PDAC) is an aggressive malignancy characterized by a complex tumor microenvironment. Ubiquitination regulates key oncogenic processes and microenvironmental remodeling; however, its cell type–specific activity and spatial organization within the PDAC microenvironment remain poorly understood.

**Materials and Methods:**

Single‐cell RNA sequencing, spatial transcriptomics, and bulk RNA‐seq datasets of PDAC were integrated for multiomics analysis. Ubiquitination activity was quantified using gene‐set scoring algorithms, followed by cell‐communication and spatial interaction analyses. Prognostic models were constructed using bulk cohorts, and key findings were validated through gene‐silencing functional assays.

**Results:**

Ubiquitination activity was significantly increased in PDAC tissues compared with adjacent normal tissues, with ductal epithelial cells and fibroblasts showing the most prominent elevation. High ubiquitination states were associated with enhanced ductal–fibroblast interactions and distinct spatial patterns linked to invasion‐associated tumor regions. Integration of ubiquitination‐associated gene signatures identified a robust prognostic model, highlighting an extracellular matrix–related factor consistently overexpressed in tumors and associated with poor survival. Functional assays demonstrated that suppression of this factor inhibited proliferation, migration, invasion, and survival of pancreatic cancer cells.

**Conclusion:**

Ubiquitination organizes ductal–fibroblast crosstalk within the PDAC microenvironment and links spatial tumor ecology with disease aggressiveness and patient prognosis. Targeting ubiquitination‐associated microenvironmental programs may offer new strategies for prognostic stratification and therapeutic intervention.

## 1. Introduction

Pancreatic ductal adenocarcinoma (PDAC) is known for its aggressive nature and extremely poor prognosis. Although it is relatively uncommon, more than 80% of patients are diagnosed at a late stage or after the cancer has spread to other organs, mainly due to the absence of early symptoms and the challenges in making an accurate diagnosis [1, 2]. For patients with advanced PDAC, surgical resection is often not an option, thereby severely limiting treatment strategies and contributing to the dismal outcomes [3]. Only approximately 20% of patients are diagnosed at an early stage, when surgical intervention is feasible [4]. However, even among those who undergo successful resection, approximately 70% experience recurrence within 2 years, with a 5‐year survival rate of less than 10% [5, 6]. These statistics highlight the urgent need for a deeper understanding of the tumor microenvironment (TME) and factors influencing the prognosis of PDAC.

Ubiquitination, a key protein modification process, delivers target proteins to the proteasome for degradation through ubiquitin tagging [7]. In cancer cells, ubiquitination plays a pivotal role in regulating essential proteins involved in cell cycle control, cellular proliferation, DNA repair, and apoptosis [8, 9]. Dysregulation of this process can drive tumor progression by altering the stability of key oncogenes and tumor suppressors.

Recently, increasing attention has been directed toward the role of ubiquitination within the TME of PDAC [10]. Ubiquitination not only modulates intrinsic cancer cell pathways but also regulates various components of the TME, which significantly influences tumor progression and response to therapy. For example, UBR5, an E3 ubiquitin ligase, has been found to promote glycolytic metabolism and tumor growth in PDAC by downregulating the metabolic repressor FBP1 and destabilizing C/EBP*α* [11]. In addition, USP25 acts as a deubiquitinating enzyme that promotes HIF‐1‐driven metabolic reprogramming and has been suggested as a potential therapeutic target to influence immune escape and prognosis in PDAC [12]. These findings provide a theoretical basis for the development of novel therapeutic strategies targeting the ubiquitination mechanism. Understanding the intricate regulatory mechanisms of ubiquitination in PDAC tumorigenesis and progression could open new avenues for enhancing the efficacy of immunotherapy and improving patient outcomes. By unraveling these mechanisms, it is possible to develop novel therapeutic strategies that could positively impact prognosis. However, there is still a lack of systematic studies on how ubiquitination affects immune response and prognosis by remodeling the TME in PDAC, especially the lack of mechanistic interpretation integrating ubiquitin‐related molecule expression profiles in association with immune infiltration and prognosis. The present study is aimed at revealing the interaction patterns between key ubiquitin regulatory factors and the immune microenvironment in PDAC through the comprehensive analysis of multiomics data, providing a theoretical basis for the discovery of new therapeutic targets and prognostic biomarkers.

## 2. Materials and Methods

### 2.1. Data Sources

This study integrated single‐cell sequencing datasets curated from the Gene Expression Omnibus (GEO) database (https://www.ncbi.nlm.nih.gov/geo/). Specifically, the analyzed datasets included GSE212966, which comprises single‐cell transcriptomic profiles from three noncancerous pancreatic tissues and six pancreatic cancer samples; GSE197177, containing data from one normal pancreatic tissue; and GSE235315, which includes single‐cell data from seven pancreatic cancer tissues. In addition, spatial transcriptomics data from pancreatic cancer tissue sections were obtained from GSE235315.

A total of 78 ubiquitination‐related genes were retrieved from the Gene Ontology (GO) database (https://geneontology.org/) for subsequent analyses. Bulk RNA sequencing (RNA‐seq) data of pancreatic cancer samples were downloaded from The Cancer Genome Atlas (TCGA) cohort through the UCSC Xena platform (https://xena.ucsc.edu/), providing transcriptomic profiles for 183 samples along with corresponding survival information for downstream prognostic analyses.

### 2.2. Single‐Cell Sequencing Data Quality Control, Dimensionality Reduction Clustering, and Cell Type Identification

The raw single‐cell sequencing data were processed using the Seurat package (Version 4.3.0) [13, 14]. Doublets were removed using the DoubletFinder package (Version 2.0.4), ensuring that each gene expression profile accurately represented individual cells and minimizing false positive signals [15]. Cells were filtered to retain those expressing 500–9000 genes and with mitochondrial gene content < 15%. Data normalization, identification of 2000 highly variable genes, scaling, and principal component analysis (PCA) were performed using Seurat′s standard pipeline. Batch effects were corrected using Harmony (Version 1.2.0) [16]. Cell clustering was conducted with FindNeighbors and FindClusters (resolution = 0.5) and visualized via Uniform Manifold Approximation and Projection (UMAP). Cell types were annotated based on known pancreatic markers from the CellMarker database. Differentially expressed genes (DEGs) for each cluster were identified using the Wilcoxon test in FindAllMarkers. Clusters were grouped into epithelial, stromal, and immune compartments, and subsetted for further refinement through repeated QC and reclustering. Marker expression was visualized using bubble plots, enabling precise cell‐type annotation across the integrated dataset.

### 2.3. Ubiquitination Score Based on Five Algorithms

A set of ubiquitin‐related genes was sourced from the GO database. To evaluate ubiquitination levels within the single‐cell dataset, five distinct scoring algorithms were utilized: AUCell, UCell, Singscore, ssGSEA, and AddModuleScore [17–20]. These complementary methods provided a robust framework for assessing ubiquitination activity across diverse cell populations. Specifically, AUCell identifies cells actively expressing a gene set by calculating the area under the curve (AUC) of ranked gene expression; UCell is a rank‐based method optimized for scalability in large single‐cell datasets; Singscore provides a simple and interpretable rank‐based single‐sample scoring; ssGSEA (single‐sample Gene Set Enrichment Analysis) estimates pathway enrichment per sample by integrating expression ranks; and AddModuleScore, implemented in Seurat, computes average expression of gene sets subtracted by aggregated control features. The use of multiple scoring approaches ensured consistency and robustness in estimating ubiquitination‐related activity patterns across cell types. To integrate scores across methods, all outputs were first standardized using the scale function and then normalized to ensure comparability. This approach improved the consistency and interpretability of ubiquitination level assessments across cell types.

### 2.4. CellChat Analysis, Enrichment Analysis and Copy Number Variation (CNV) Analysis Under the Difference of Ubiquitination Level

Ductal cells and fibroblasts were classified into high and low ubiquitination groups based on the median ubiquitination score. The CellChat package (Version 1.6.1) was employed to investigate intercellular communication [21]. This comprehensive analysis included the identification of ligand‐receptor pairs, reconstruction of signaling pathways, quantification of communication strength, and network construction, which were visualized to uncover differences in cell interactions between ductal cells and fibroblasts with varying levels of ubiquitination.

To explore functional differences between ductal cells and fibroblasts under different ubiquitination levels, the FindMarkers function was applied for differential gene expression analysis. Genes were categorized as upregulated or downregulated based on their avg_log2FC values. KEGG pathway enrichment analysis was conducted using the CompareCluster function in the ClusterProfiler package (Version 4.8.3) [22]. This analysis identified significantly enriched KEGG pathways for both upregulated and downregulated genes, with statistical significance calculated for each pathway [23]. By comparing the pathway enrichment across these gene groups, we identified the most relevant biological pathways influenced by varying ubiquitination levels.

For the assessment of genomic alterations, the CopyKAT package (Version 1.1.0) was utilized to analyze CNVs in epithelial cells [24]. This analysis provided CNV profiles for both cancerous and normal epithelial cells, shedding light on genomic instability related to changes in ubiquitination levels.

### 2.5. Spatial Transcriptome Sequencing (stRNA‐seq) Data Processing and Metabolic Analysis

Spatial transcriptomic data were processed using the Seurat package. Genes with low expression (detected in < 10 spots) and ribosomal or mitochondrial genes were removed. Data normalization was performed using SCTransform, followed by dimensionality reduction (PCA) and clustering (FindNeighbors and FindClusters). Clusters were visualized via DimPlot (UMAP) and their spatial distribution was mapped using SpatialDimPlot [25]. To further explore the functional characteristics of the identified cell populations, metabolic activity related to ubiquitination‐associated gene expression was assessed. Metabolic pathway analysis was performed using the scMetabolism package (Version 0.2.1), with the KEGG database as a reference. The AUCell method was employed to quantify the activity of metabolic pathways across the various cell clusters [26].

### 2.6. Deconvolution Analysis and MistyR

Annotated single‐cell transcriptome data were used to extract gene expression profiles and cell type information. A Reference object was created to infer the distribution of cell types within the spatial transcriptome data. Using the GetTissueCoordinates function, the spatial coordinates for tissue sections were extracted from the processed data. A gene expression matrix was then generated, and the unique molecular identifier (UMI) count for each spatial point was calculated. Finally, SpatialRNA objects were constructed. The RCTD package was used to perform cell type deconvolution, and the results were integrated with the spatial transcriptomic data after clustering, providing insights into the distribution and proportions of different cell types within tissue sections [27, 28].

For further analysis of intercellular communication, the mistyR package (Version 1.6.1) was applied to the deconvoluted spatial transcriptomic data. This method identified the spatial distribution and interactions between various cell types in pancreatic cancer tissues. The analysis revealed spatial dependencies in cell communication, enhancing our understanding of the TME′s structural and functional characteristics [29].

### 2.7. Spatial Characterization and Trajectory Analysis of ANAPC13^+^ Ductal Cells and HECW2^+^ Fibroblasts

Based on the expression patterns of ANAPC13 and HCEW2, ductal cells and fibroblasts were classified into positive and negative groups. To investigate the spatial distribution of ANAPC13^+^ ductal cells and HCEW2^+^ fibroblasts within tissue sections, we utilized the SpatialFeaturePlot function to visualize these features. This analysis is aimed at providing a comprehensive understanding of how these cell types are distributed in tissues and their potential roles within the TME. To further explore the spatial relationships between positive and negative cells and other cell populations, correlation matrices were constructed using the plotCorrelationMatrix function from the SPOTlight package [30]. Additionally, the plotInteractions function was employed to visualize interaction networks, enabling a clearer depiction of how these cells interact with their surroundings.

High‐infiltration zones of ANAPC13^+^ ductal cells and HCEW2^+^ fibroblasts were identified, and the corresponding cell clusters were extracted for further analysis. The Monocle package was applied to perform pseudo‐temporal analysis, reconstructing the differentiation and developmental trajectories of these cell populations [31–33].

### 2.8. Prognostic and Immunological Relevance of ANAPC13^+^ Ductal Cells and HECW2^+^ Fibroblasts

The single‐cell transcriptome data were processed using the Seurat package. Based on the expression of ANAPC13 or HCEW2, ductal cells and fibroblasts were divided into positive and negative groups. Marker genes unique to each group were identified through the FindMarkers function. For the positively expressed groups, the infiltration levels of corresponding marker genes were analyzed in bulk RNA‐seq datasets, which facilitated the classification of samples into high‐ and low‐infiltration categories.

To evaluate the functional activity of marker gene sets, the ssGSEA method was employed. Enrichment analysis provided insights into their biological roles across different samples [20]. Survival data were imported, preprocessed using the fread function, and integrated with enrichment results. Prognostic differences based on infiltration levels were assessed using the survival and survminer packages. Kaplan–Meier (KM) survival curves, generated via the ggsurvplot function, enabled a clear comparison of survival outcomes between the high‐ and low‐infiltration groups, emphasizing the prognostic relevance of these genes.

Additionally, we analyzed the relationship between ANAPC13 expression in ductal cells, HCEW2 expression in fibroblasts, and tumor immune escape scores. Immune‐related metrics were utilized to explore potential immune evasion mechanisms within the TME, offering valuable guidance for future immunotherapeutic interventions.

### 2.9. Acquisition of Common Differential Genes, Construction and Evaluation of Machine Learning Models, and Survival Analysis

Differential expression analysis was conducted on tumor and normal samples from TCGA RNA‐seq data using the DESeq2 package, generating a list of DEGs [34]. Simultaneously, the FindMarkers function in the Seurat package was applied to identify DEGs between ANAPC13‐positive and ANAPC13‐negative ductal cells, as well as between HCEW2‐positive and HCEW2‐negative fibroblasts, based on single‐cell sequencing data. The DEGs identified from these analyses were intersected with the DEGs from TCGA, yielding 16 common differential genes.

These 16 DEGs were used to develop prognostic models via the mime R package, which systematically evaluates 101 machine learning algorithms including Cox‐based models (e.g., LASSO‐Cox, Ridge), tree‐based models (e.g., Random Survival Forest, Gradient Boosting), and support vector machine methods. The TCGA dataset served as the training cohort, and GSE85916 was used for external validation. Model performance was assessed using the concordance index (C‐index), and the algorithm with the highest validation C‐index was selected as the final model. Risk scores were computed for each patient using the rs_predict function, and patients were stratified into high‐risk (top 55%) and low‐risk (bottom 45%) groups. KM analysis demonstrated significant survival differences, highlighting the model′s prognostic value.

### 2.10. Association Analysis Between LAMA3 Gene Expression and Survival Status

Machine learning analysis identified six key genes linked to the lasso + survivalSVM model. Among them, LAMA3 was prioritized for detailed investigation due to its strong biological relevance and its notable connection to ubiquitination

To examine LAMA3 expression in pancreatic cancer, we used gene expression data from the curated TCGA dataset and RNA‐seq data from the PanCanAtlas. The Wilcoxon rank‐sum test was applied to compare LAMA3 expression levels between normal and tumor samples, as well as across various tumor grades. For paired normal‐tumor samples, paired Wilcoxon tests (wilcox.test) were performed after processing paired sample files

LAMA3 expression data and corresponding survival information were retrieved using the getPanGeneTime function. Expression levels were divided into four quartiles (Q1–Q4), with Q1 representing the highest expression levels and Q4 the lowest. Survival data were integrated with these quartiles to construct a comprehensive dataset for analysis. The chi‐square test was utilized to evaluate the association between LAMA3 expression levels and survival outcomes by comparing survival distributions across quartiles.

Survival metrics, including progression‐free interval (PFI), overall survival (OS), disease‐specific survival (DSS), and disease‐free interval (DFI), were further analyzed. Survival durations were converted to years (365 days), and only samples with PFI values greater than zero were included. The surv_cutpoint function determined the optimal LAMA3 expression threshold based on PFI, categorizing samples into high‐ and low‐expression groups. KM survival analysis was performed to visualize survival trends, and the survdiff function assessed statistical differences between these groups.

To enhance the depth of analysis, we stratified gene expression levels into quartiles (Q1, Q2, Q3, and Q4) and examined survival outcomes, including OS, DSS, DFI, and PFI, across these groups. Differences in survival outcomes between quartiles were assessed using chi‐square tests, whereas KM curves were generated to visually depict survival trends. To further evaluate the prognostic relevance of LAMA3 expression, univariate Cox regression analysis was conducted, yielding hazard ratios (HR) with corresponding 95% confidence intervals (CI) [35].

Sequential Cox regression analyses were applied across multiple datasets. Datasets with HR CI upper limits exceeding 100 were excluded to ensure reliability. For a comprehensive assessment of LAMA3′s prognostic impact, a meta‐analysis was performed using the meta package in R. A random‐effects model was employed to calculate the pooled HR, integrating results from various datasets. This approach provided a robust evaluation of LAMA3′s prognostic value across different study populations.

### 2.11. Gene Set Enrichment Analysis (GSEA)‐Hallmark Analysis and GO Functional Enrichment Analysis

To identify key pathways enriched within the gene expression data, GSEA was carried out using the clusterProfiler package, leveraging hallmark gene sets from the Molecular Signature Database (MSigDB) [20, 22]. Enrichment scores were calculated for each pathway, quantifying the extent to which specific gene sets were overrepresented at the extremes of the ranked gene list. This analysis provided valuable insights into the biological significance underlying the observed expression patterns.

In addition, GO functional enrichment analysis was performed, also utilizing the clusterProfiler package [36]. This analysis is aimed at further exploring the functional roles of the identified genes. The enriched GO terms were categorized into three major domains: biological processes, cellular components, and molecular functions. The enrichment results were visualized as bar plots, where the enrichment size and statistical significance highlighted the importance of each gene set.

### 2.12. Genomic Alteration and Immune Infiltration Analysis of LAMA3 in PDAC

To investigate the genomic alteration characteristics of LAMA3 in PDAC, somatic mutation data from the TCGA cohort were analyzed. The mutation landscape of LAMA3 was visualized using lollipop plots to display the distribution and types of mutations along the protein sequence. To further explore genomic alteration patterns associated with LAMA3 expression, PDAC samples were stratified into LAMA3‐high and LAMA3‐low groups based on the median expression level. Mutation profiles of common driver genes and copy number alterations (CNAs), including genomic gains and losses across chromosomal regions, were visualized using oncoprint‐based approaches. To assess the potential association between LAMA3 alteration status and the tumor immune microenvironment, immune cell infiltration data for TCGA PDAC samples were obtained from the TIMER2.0 platform. Samples were divided into LAMA3 mutant and wild‐type groups, and differences in immune cell infiltration between the two groups were evaluated using the Wilcoxon rank‐sum test. Immune cell types showing significant differences were visualized using heat maps.

### 2.13. Cell Culture and Transfection

In this study, two pancreatic cancer cell lines, SW1990 and PANC‐1, were used. These cell lines were obtained from the cell bank of the Central Laboratory of the Affiliated Hospital of Southwest Medical University. Cells were cultured in RPMI‐1640 medium (HyClone) supplemented with 10% fetal bovine serum (FBS, HyClone), 100 U/mL penicillin, and 100 *μ*g/mL streptomycin (Thermo Fisher Scientific) at 37°C in a humidified atmosphere with 5% CO_2_.

For transient transfection, Lipofectamine 3000 reagent (Invitrogen, Carlsbad, California, United States) was used according to the manufacturer′s instructions. Nontargeting siRNA was transfected into the negative control (NC) group, whereas siRNA targeting LAMA3 (siRNA‐LAMA3) was transfected into the experimental group. Cell density was adjusted to 50%–70% confluency before transfection to ensure optimal transfection efficiency. After 48 h of transfection, cells were harvested for subsequent experiments to investigate the effects of LAMA3 knockdown on pancreatic cancer cell functions.

### 2.14. Quantitative Real‐Time PCR (qRT‐PCR)

To measure LAMA3 mRNA expression levels, total RNA was extracted from SW1990 and PANC‐1 cells using TRIzol reagent (Invitrogen). The extracted RNA was reverse‐transcribed into complementary DNA (cDNA) using the PrimeScript RT Reagent Kit (Takara). Real‐time PCR was performed using SYBR Green Master Mix (Takara) on an ABI 7500 Real‐Time PCR System. The reaction conditions were as follows: 95°C for 30 s for predenaturation, followed by 40 cycles of 95°C for 5 s and 60°C for 30 s.

GAPDH was used as the internal reference gene, and the relative expression of target genes was calculated using the 2^−*ΔΔ*Ct^ method.

### 2.15. Cell Counting Kit‐8 (CCK‐8) Proliferation Assay

Cell proliferation was assessed using the CCK‐8 (Dojindo). After 24 h of transfection, SW1990 and PANC‐1 cells were seeded into 96‐well plates at a density of 1500 cells per well in 200 *μ*L of complete medium. At 24, 48, 72, and 96 h, 10 *μ*L of CCK‐8 reagent was added to each well and incubated at 37°C for 2 h. The optical density (OD) was measured at 450 nm using a microplate reader to evaluate the relative proliferation levels.

### 2.16. Colony Formation Assay

To assess the colony‐forming ability of pancreatic cancer cells, SW1990 and PANC‐1 cells were seeded into 6‐well plates at a density of 500 cells per well after transfection. Cells were cultured for 2 weeks, with the medium changed every 3 days. Colonies were fixed with 4% paraformaldehyde and stained with 0.1% crystal violet for 30 min. Excess dye was gently washed off, and the colonies were counted and photographed under a light microscope.

### 2.17. Wound Healing Assay

The migration ability of pancreatic cancer cells was evaluated using the wound healing assay. Transfected SW1990 and PANC‐1 cells were seeded in 6‐well plates and cultured until 90% confluence. A sterile 200 *μ*L pipette tip was used to create a straight‐line scratch in the cell monolayer. The detached cells were washed away with PBS twice, and serum‐free medium was added to each well. Images of the wound area were captured at 0 h and 48 h using an Olympus inverted microscope. The wound closure rate was quantified using ImageJ software to assess cell migration.

### 2.18. Transwell Invasion Assay

The invasive ability of pancreatic cancer cells was evaluated using Transwell chambers (8 *μ*m pore size, Corning) coated with Matrigel. Transfected SW1990 and PANC‐1 cells were resuspended in serum‐free medium and seeded into the upper chamber at a density of 2 × 10^4^ cells per well. Complete medium with 10% FBS was added to the lower chamber as a chemoattractant. After 24 h of incubation at 37°C, the noninvaded cells in the upper chamber were removed with a cotton swab. The invaded cells on the lower side of the membrane were fixed with 4% paraformaldehyde and stained with 0.1% crystal violet. Invaded cells were counted under a microscope in five random fields to quantify invasion ability.

### 2.19. Flow Cytometry for Apoptosis Analysis

The apoptotic rate of SW1990 and PANC‐1 cells was measured using an Annexin V‐FITC/PI Apoptosis Detection Kit (BD Biosciences). After 48 h of transfection, cells were harvested and washed twice with PBS. According to the manufacturer′s protocol, cells were stained with Annexin V‐FITC and PI in the dark at room temperature for 15 min. Apoptosis was analyzed using a BD FACSCanto II flow cytometer, and the data were processed using FlowJo software to determine the percentages of early and late apoptotic cells.

### 2.20. Statistical Analysis

The statistical analyses were conducted using R Version 4.2.2, 64‐bit, along with its support packages. The nonparametric Wilcoxon rank sum test was employed to assess the relationship between two groups for continuous variables. Spearman correlation analysis was conducted to examine correlation coefficients. A significance level of *p* < 0.05 was considered statistically significant for all statistical investigations.

All experiments were performed in triplicate, and the data are presented as mean ± standard deviation (mean ± SD). Statistical analysis was conducted using GraphPad Prism 8.0 software. Differences between two groups were assessed using a two‐tailed *t*‐test, and *p* values < 0.05 were considered statistically significant.

## 3. Results

### 3.1. Identification of Cell Types in Adjacent Normal and Tumor Tissues of PDAC

Single‐cell RNA sequencing (scRNA‐seq) data from PDAC tumor tissues and adjacent normal tissues were processed and analyzed, encompassing a total of 89,208 cells. These included 63,509 cells from tumor tissues and 25,699 cells from adjacent normal tissues. To minimize artifacts introduced by doublets during sequencing, doublet removal was performed, reducing the dataset to 57,230 cells, comprising 39,429 tumor cells and 17,801 normal cells.

**Figure 1:**
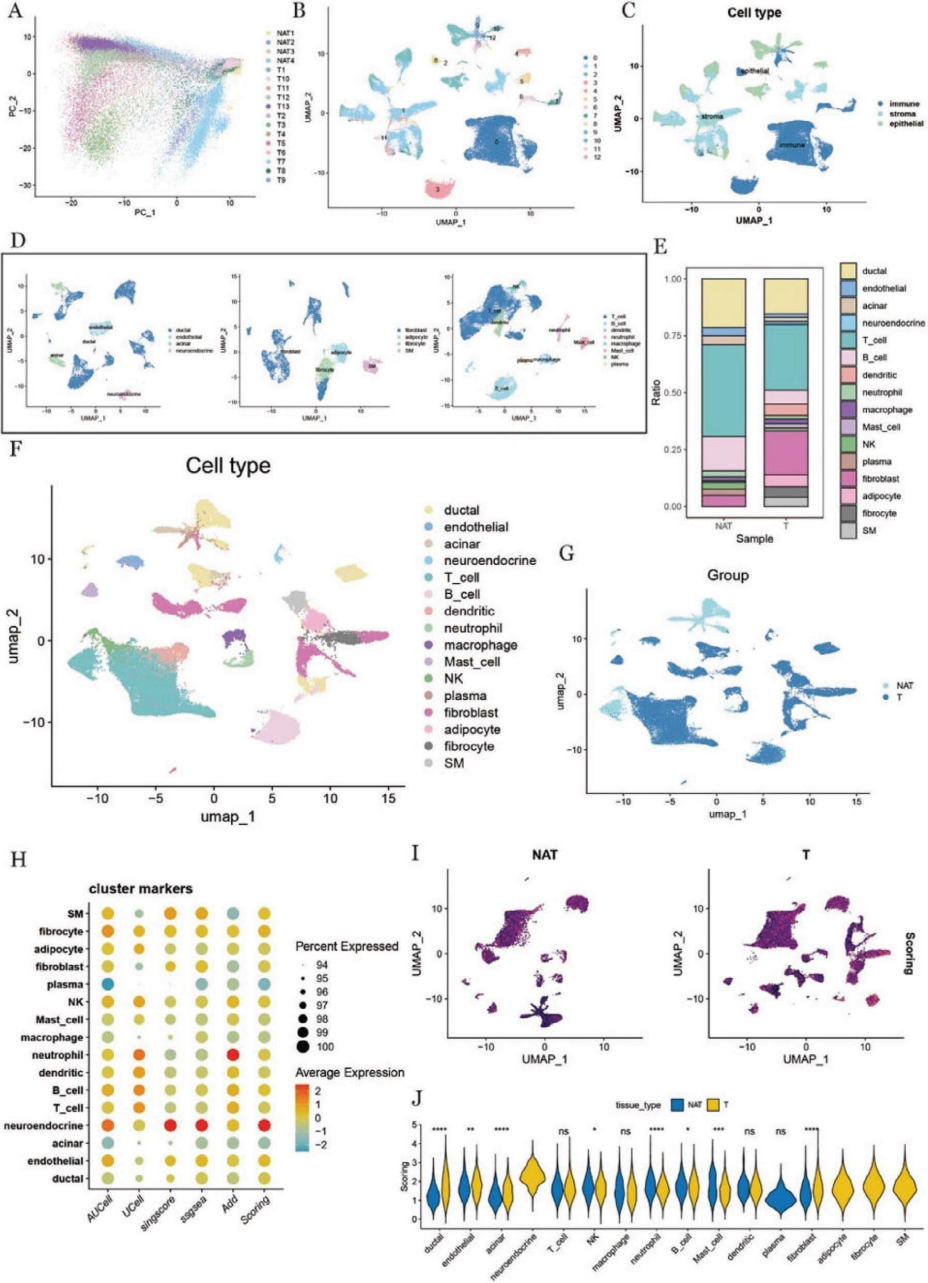
Cell type recognition and ubiquitination score. (A) Sample distribution map based on principal component analysis (PCA), with different colors representing different samples, a total of 17 samples. (B) UMAP after dimensionality reduction clustering, with a total of 13 cell clusters. (C) UMAP map of preliminary identification of cell types, showing the three cell types. (D) From left to right, annotated UMAP map of subsets of epithelial cells, stromal cells, and immune cells. (E) Proportion diagram of cell types, which were displayed in adjacent normal tissues and tumor tissues. (F) UMAP after the final identification of cell types, a total of 16 cell types. (G) UMAP of tissue type. (H) Quantification of ubiquitination level in different cell types based on five different scoring methods. (I) Expression profile of ubiquitin‐related genes based on UMAP dimensionality reduction, grouped by tissue type. (J) Violin plot of the difference in ubiquitination scores between adjacent normal tissues and tumor tissues. With “ ^∗^” said *p* values < 0.05, *p* values < 0.01, “ ^∗∗^” said “ ^∗∗∗^,” said *p* values < 0.001, “ ^∗∗∗∗^,” said p values < 0.0001, an asterisk, the *p* value is smaller, the more significant difference.

The scRNA‐seq data underwent multiple preprocessing steps, including quality control, data normalization, creation of a normalized expression matrix, identification of highly variable genes, data scaling, and dimensionality reduction. PCA was performed to map the distribution of cells, which was visualized in a two‐dimensional space (Figure 1A). To improve the accuracy of cell‐type classification, batch effects were corrected, and dimensionality reduction clustering identified 12 distinct cell clusters, visualized using UMAP (Figure 1B).

Using marker genes specific to pancreatic tissues, sourced from the CellMarker database, cells were initially grouped into three main categories: epithelial cells, stromal cells, and immune cells (Figure 1C). Subsequent clustering and marker gene analyses further subdivided these groups into specific cell types: epithelial cells (ductal epithelial cells, endothelial cells, acinar cells, and neuroendocrine cells), stromal cells (fibroblasts, vascular smooth muscle cells, and adipocytes), and immune cells (T cells, B cells, NK cells, dendritic cells, neutrophils, macrophages, mast cells, and plasma cells). A total of 16 distinct cell types were identified (Figure 1D). Notably, fibroblasts were significantly enriched in tumor tissues compared with adjacent normal tissues, indicating their potential role in PDAC progression (Figure 1E). To further highlight these findings, UMAP visualizations were generated to depict the overall distribution and abundance of all cell types (Figure 1F), as well as their specific distributions within tumor and normal tissues (Figure 1G).

### 3.2. Quantification of Ubiquitination Level in the TME of Pancreatic Cancer

To examine differences in ubiquitination levels between tumor tissues and adjacent normal tissues, a panel of 78 ubiquitination‐related genes was analyzed. Five scoring methods—AUCell, UCell, singscore, ssGSEA, and AddScoring—were applied to estimate the relative activity of this gene set across various cell types (Figure 1H).

UMAP was used to visualize ubiquitination scores within tumor and normal tissues, revealing distinct patterns across the TME (Figure 1I). Statistical analysis demonstrated that ductal epithelial cells and fibroblasts exhibited significantly elevated ubiquitination scores in tumor tissues compared with adjacent normal tissues, highlighting their potential involvement in tumor biology (Figure 1J).

### 3.3. Ductal Epithelial Cells and Fibroblasts Under Differences in Ubiquitination Levels

**Figure 2:**
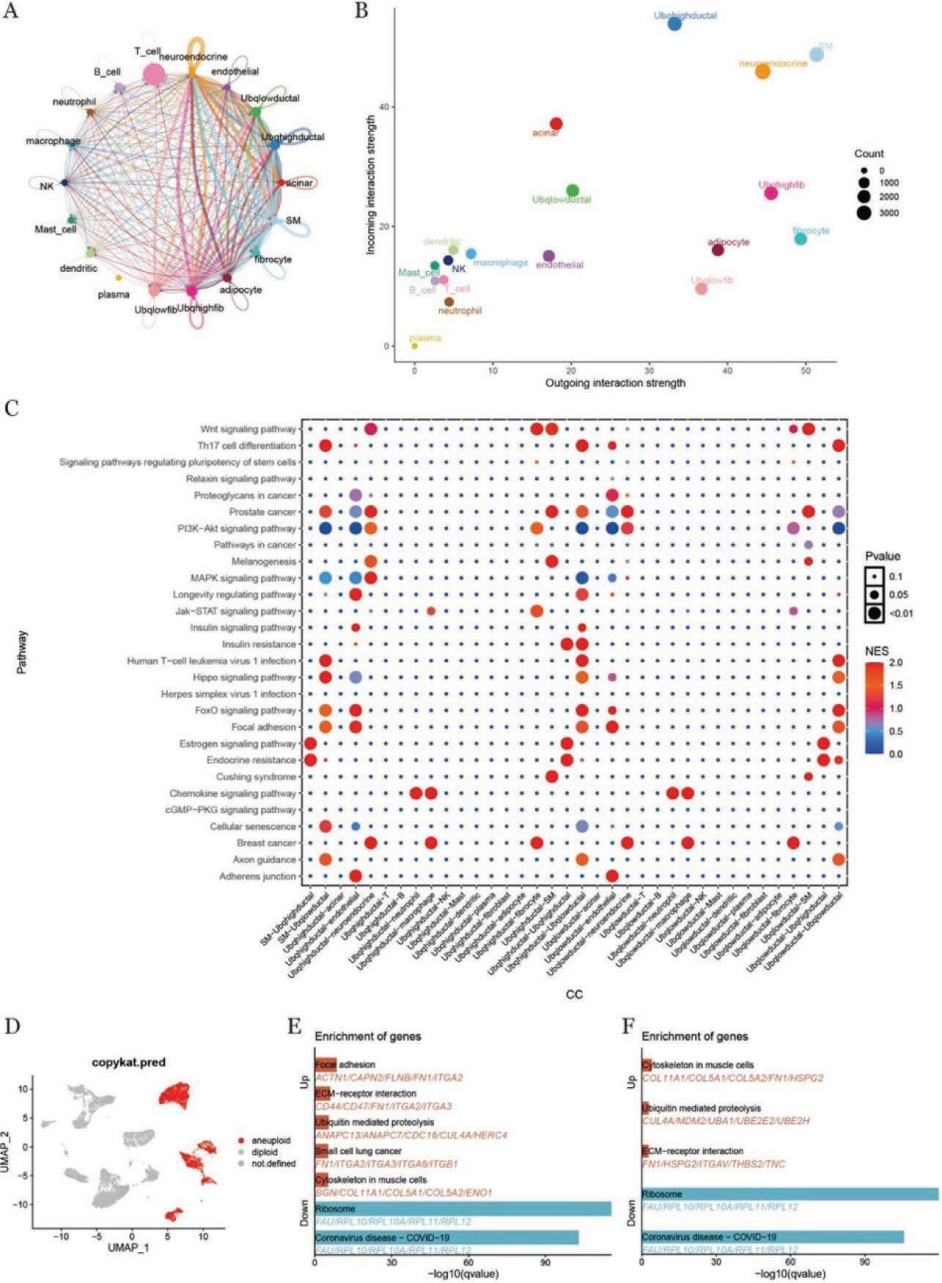
Cell communication, copy number variation analysis, and enrichment analysis under differences in ubiquitination levels. (A) Chordogram of cell communication, showing the number and strength of interactions. (B) Scatter plot of signaling roles between cells, each point represents a cell type, and the horizontal and vertical axes represent the signaling ability of that cell type to send and receive signals, respectively. (C) Bubble plot of signaling pathways between ductal cells and other cells under different ubiquitination levels. (D) Different cell copy number states are shown by UMAP dimensionality reduction diagram. Different color schemes were used to distinguish cells with normal from abnormal copy numbers, with red as abnormal. (E, F) Enrichment analysis of differential genes in ductal cells and fibroblasts under different ubiquitination levels; the horizontal axis represents the enrichment significance of each pathway, the vertical axis represents the name of the differential pathway, and the enriched genes are marked below each pathway.

To explore the transcriptional correlates of ubiquitination in intercellular signaling, ductal epithelial cells and fibroblasts from tumor tissues were divided into high‐ and low‐ubiquitination groups based on the median score. Cell–cell communication analysis using CellChat suggested increased predicted interaction strength and frequency between ductal cells and fibroblasts in the high‐ubiquitination group (Figure 2A), trend also reflected in ligand–receptor signaling input and output patterns (Figure 2B). Further pathway‐level comparison indicated differences in predicted signaling potentials between ductal cells with high versus low ubiquitination scores (Figure 2C). Concurrently, CNV analysis was conducted for epithelial cells, with UMAP visualizations illustrating the distribution of cell populations based on CNV. Results indicated that ductal epithelial cells constituted the majority of the population with significantly elevated copy numbers (Figure 2D).

To explore functional differences, we conducted enrichment analysis of DEGs in ductal cells and fibroblasts with high‐ and low‐ubiquitination levels (Figures 2E,F). The analysis revealed consistent upregulation and downregulation of specific pathways in both cell types, with the ubiquitin‐mediated proteolysis pathway being notably upregulated in both ductal cells and fibroblasts. This suggests that, despite potential variations in the underlying mechanisms, ubiquitination regulation plays a critical and shared role in tumor progression. The key genes associated with this pathway in ductal cells included ANAPC3, ANAPC7, CDC16, CUL4A, and HERC4, whereas in fibroblasts, the top genes were CUL4A, MDM2, UBA1, UBE2E2, and UBE2H. Building on these findings, we decided to focus on ANAPC3 in ductal epithelial cells and HECW2 in fibroblasts to further investigate the impact of ubiquitination levels on tumor cells and their surrounding microenvironment.

### 3.4. Spatial Clustering Identifies Metabolically Active Niches Within the PDAC Microenvironment

**Figure 3:**
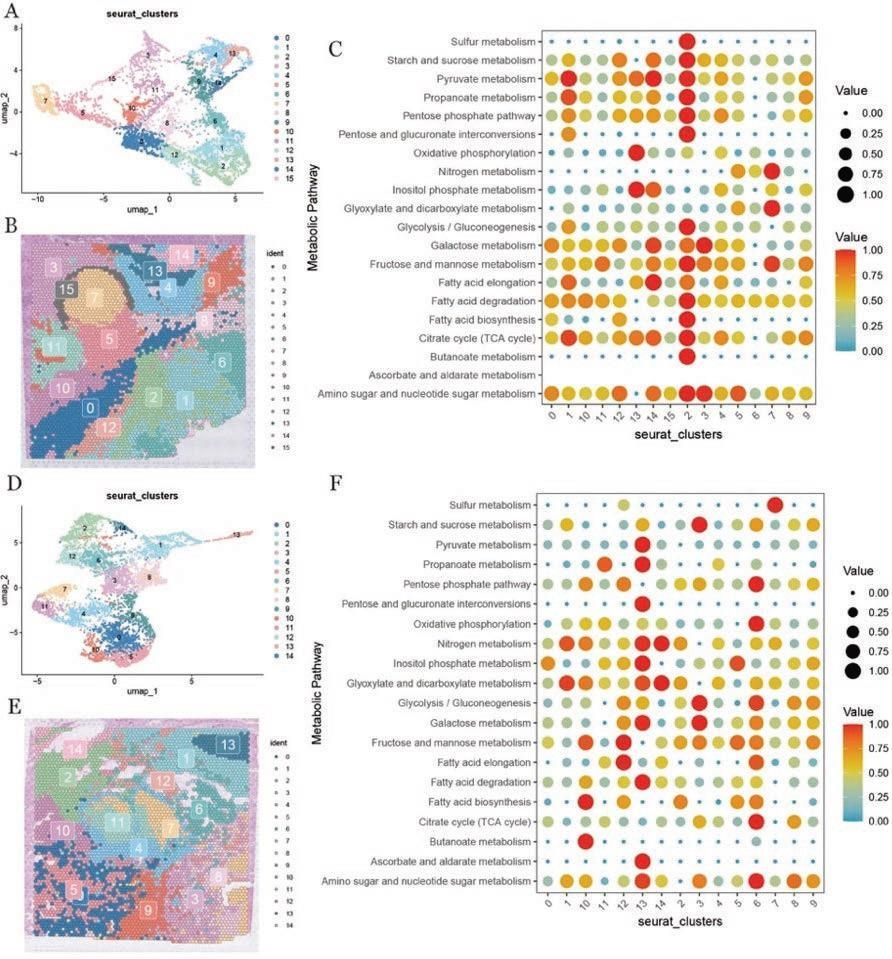
Spatial transcriptome data clustering and metabolic analysis. (A, D) UMAP plots showing the clustering results of spatial transcriptome data; (B, E) showing the spatial location of cell clusters in tissue sections; (C, F) bubble plots of metabolic pathways in cell clusters, with larger dots and darker colors indicating higher expression levels of this pathway.

Dimensionality reduction and clustering were performed on the spatial transcriptomic data from two PDAC tissue sections. The resulting clusters were visualized using UMAP to illustrate their relationships in a two‐dimensional space, revealing 16 and 15 distinct clusters in the two sections, respectively (Figure 3A,D). To better illustrate the spatial organization of cell populations in the tissue sections, we visualized their spatial distributions; spatial cluster maps were generated, in which each cluster was represented by a distinct color corresponding to its cluster identity (Figure 3B,E) [37]. This visualization enabled clear delineation of the spatial distribution of cell populations across tissue sections.

To further investigate functional heterogeneity among spatially defined clusters, metabolic pathway activity was assessed based on spatial transcriptomic profiles. The resulting metabolic activity scores were visualized across clusters, revealing pronounced metabolic activation in Cluster 2 of the first tissue section (Figure 3C), as well as in Clusters 6 and 13 of the second section (Figure 3F). These metabolically active clusters were spatially restricted to specific tissue regions, suggesting localized metabolic reprogramming within the TME.

### 3.5. Spatial Deconvolution Reveals Ubiquitination‐Associated Ductal–Fibroblast Crosstalk Across Multipl0065 Spatial Scales

**Figure 4:**
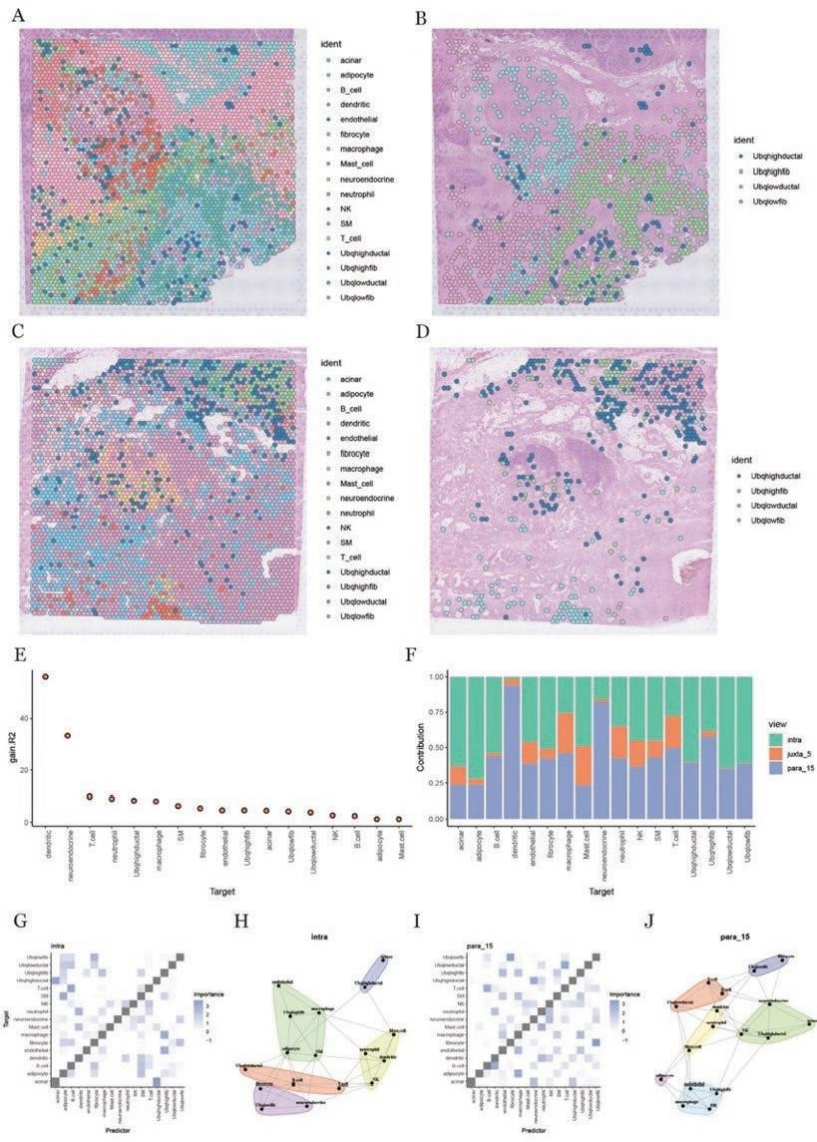
Deconvolution analysis of spatial transcriptome data and cell‐to‐cell interaction analysis. (A, C) Deconvolution analysis results in two different tissue sections. (B, D) Spatial distribution map of ductal cells and fibroblasts in tissue sections under different levels of ubiquitination in two different tissue sections. (E) Statistical plot of sample prediction accuracy modification. (F) Bar graph of the contribution of different views to cell‐to‐cell interactions in the mistyR model, showing the view that contributed the most to the prediction results. (G, H) Heat map and network map of cell interactions in intraview, showing the strength and pattern of interactions within the same cell type. (I, J) Heat map and network map of interaction between cells in para_15 view, showing the interaction strength and pattern between different cells.

Cell type–specific gene expression signatures derived from the annotated single‐cell dataset were used for spatial deconvolution, enabling estimation of the relative abundance of each cell type across spatial locations. The inferred spatial distributions of major cell populations were subsequently visualized, revealing distinct organizational patterns within the tissue sections (Figure 4A,C). Given their elevated ubiquitination activity, ductal cells and fibroblasts were further examined by stratifying them into high‐ and low‐ubiquitination groups. Spatial feature maps were generated separately for these populations to resolve their spatial localization and distribution within the tissue context (Figure 4B,D). This analysis highlighted heterogeneity in the spatial arrangement of ductal cells and fibroblasts associated with different ubiquitination states across the TME.

To further explore the interactions between cells and their surrounding environment, mistyR analysis was applied to assess the microenvironment at varying spatial resolutions. This approach allowed us to investigate how cell interactions influence gene expression, cellular functions, and tissue architecture. Visualization of the analysis models revealed how biologically relevant pathways and networks, particularly those involved in tumor progression and therapeutic response, were captured (Figure 4E). These findings offer valuable insights into the dynamic cellular interactions within the TME.

A bar chart was used to illustrate the contributions of different views or data sources to the final model outcomes. It was found that the “intra view” (within cells) and “para_15 view” (interactions with cells within a 15 *μ*m radius) had the most significant impact on the results (Figure 4F). This highlights the importance of analyzing both the internal microenvironment of individual cells and their interactions with neighboring cells within a 15 *μ*m distance to fully understand tumor biology. As a result, we chose to focus on these two perspectives for further investigation.

To gain deeper insights, heat maps were employed to investigate the interaction patterns between ductal cells and fibroblasts with high and low ubiquitination levels. These analyses took into account both intrapopulation interactions and those with neighboring cells within a 15 *μ*m radius (Figure 4G,I), offering fresh perspectives on cellular signaling and material exchange. Additionally, network diagrams were generated to illustrate the interactions between functional modules, both within the same spatial region and across different tissue domains (Figure 4H,J). These network visualizations provided a more holistic view of the complex cellular communication networks present within the TME, shedding light on the intricate relationships that contribute to tumor progression.

### 3.6. Spatial Mapping and Interaction Networks of ANAPC13^+^ Ductal Cells and HECW2^+^ Fibroblasts

**Figure 5:**
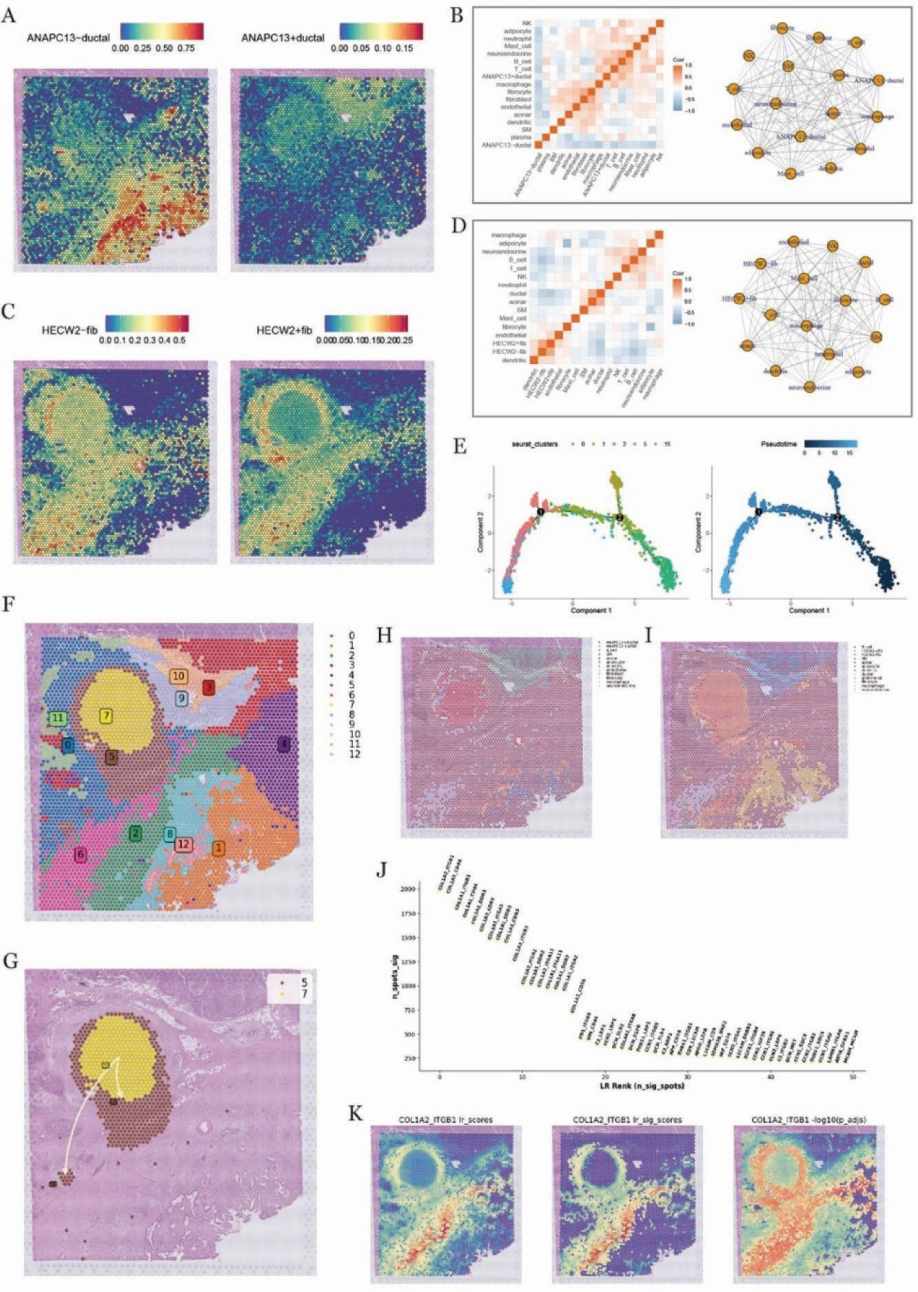
Distribution and interaction analysis of ANAPC13 and HECW2‐related cells in tissue sections. (A, C) Distribution of AnAPC13‐associated ductal cells and Hecw2‐associated fibroblasts. (B, D) Correlation plots of Anapc13‐associated ductal cells and Hecw2‐associated fibroblasts interacting with other cell types, heat maps on the left and network maps on the right. (E) Trajectory analysis of cell clusters in the region where AnAPC13‐related ductal cells and Hecw2‐related fibroblasts are located (left panel) and plots of the results of the pseudo‐timing analysis (right panel). (F) Spatial clustering analysis of the tissue section showing 16 distinct cell clusters. (G) Spatial mapping of the inferred developmental trajectory, illustrating the stepwise invasion and metastasis process of tumor cells over pseudotime. (H, I) Spatial deconvolution maps showing the localization of ANAPC13‐positive and ‐negative ductal cells, HECW2‐positive and ‐negative fibroblasts, and other cell types within the tissue section. (J) Ranking plot of significant ligand–receptor pairs identified in spatial cell communication analysis. (K) Spatial distribution of the COL1A2–ITGB1 interaction activity, including ligand–receptor scores, significant interaction scores, and adjusted significance levels.

Ductal cells were stratified into ANAPC13‐positive (ANAPC13^+^ ductal) and ANAPC13‐negative (ANAPC13^−^ ductal) subpopulations based on ANAPC13 expression in scRNA‐seq data. Similarly, fibroblasts were classified into HECW2‐positive (HECW2^+^ fib) and HECW2‐negative (HECW2^−^ fib) groups. To characterize the spatial distribution of these subpopulations, spatial deconvolution analysis was performed, generating feature maps that depicted the localization and relative abundance of ANAPC13^+^/^−^ ductal cells and HECW2^+^/^−^ fibroblasts within tissue sections (Figure 5A,C). To further explore the relationships between these subpopulations and other cellular components of the TME, correlation analyses were conducted across cell types and gene expression profiles. The resulting interaction strengths and correlation patterns were visualized using heat maps and network diagrams, illustrating the spatially resolved associations between ANAPC13^+^/^−^ ductal cells, HECW2^+^/^−^ fibroblasts, and additional cell populations (Figure 5B,D). Based on the spatial distribution patterns of positive and negative subpopulations in conjunction with clustering results, Clusters 0, 1, 2, 5, and 15 were selected for pseudo time trajectory analysis. This analysis enabled reconstruction of dynamic state transitions and differentiation trajectories of these cells across pseudo temporal progression (Figure 5E).

To further refine spatial resolution and transcriptional context, spatial transcriptomic data were reanalyzed with a focus on tumor progression–associated cellular dynamics, including invasion‐related features. After rigorous quality control and dimensionality reduction, clustering analysis identified 16 spatially distinct cell clusters (Figure 5F), which provided a framework for subsequent trajectory and localization analyses.

By integrating pseudotime reconstruction with spatial localization information, we delineated the spatial distribution of tumor cell states along inferred developmental trajectories (Figure 5G). Notably, regions exhibiting invasion‐associated transcriptional features were spatially enriched in proximity to HECW2^+^ fibroblasts (Figure 5H,I), indicating a close spatial association between these fibroblast subpopulations and tumor cells undergoing invasive state transitions. These observations suggest that HECW2^+^ fibroblasts may be preferentially localized within microenvironmental niches linked to tumor invasion.

In addition, spatial cell–cell communication analysis identified a prominent ligand–receptor interaction between COL1A2 and ITGB1 (Figure 5J). Spatial mapping of interaction activity revealed enrichment of this signaling axis within tumor regions (Figure 5K), supporting its potential involvement in extracellular matrix–mediated cell communication within the PDAC microenvironment.

### 3.7. Prognostic and Immunotherapy‐Associated Relevance of ANAPC13^+^ Ductal Cells and HECW2^+^ Fibroblasts in PDAC

**Figure 6:**
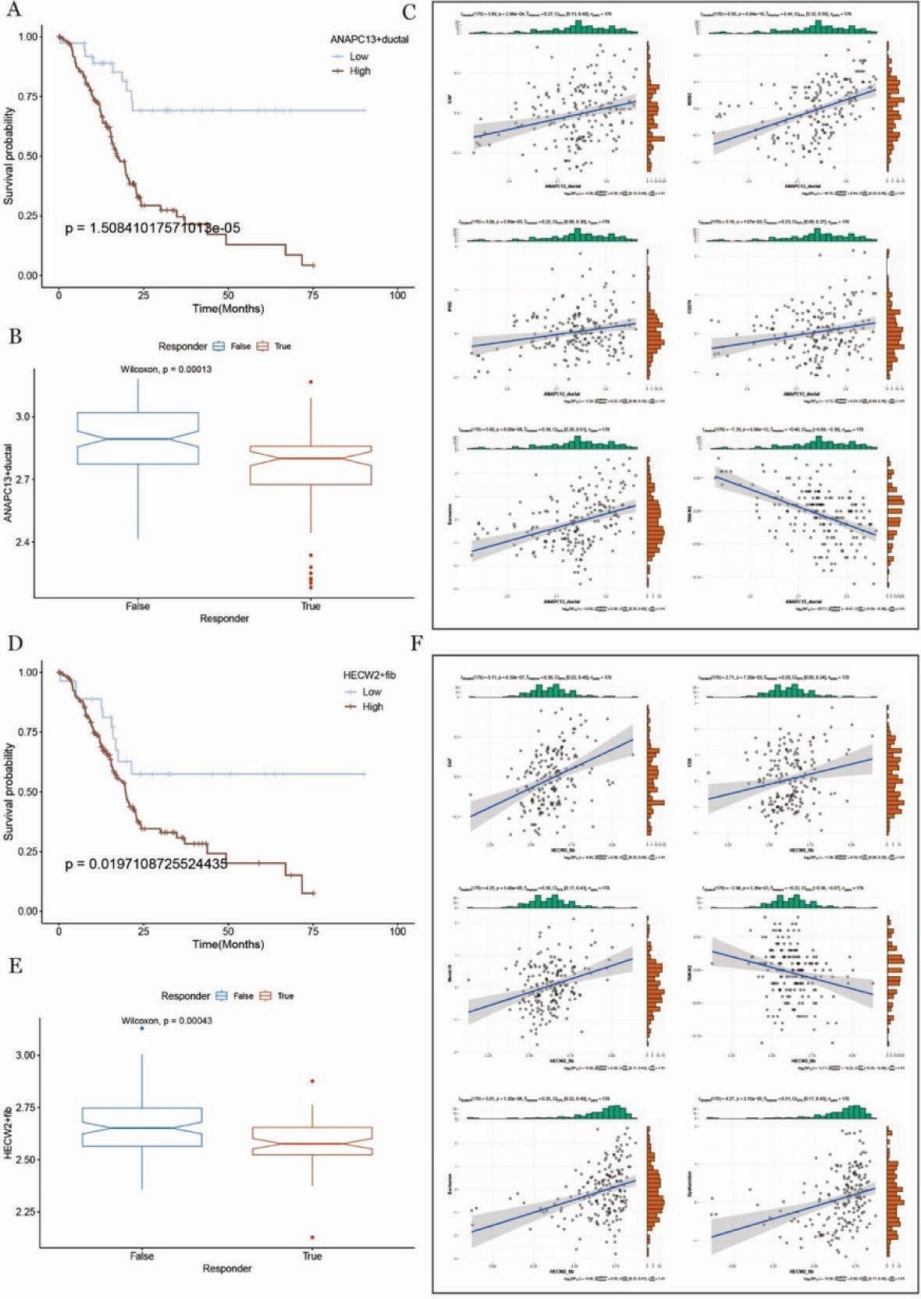
HECW2 Prognosis and immunotherapy in ductal cells ANAPC13 and fibroblasts. (A, D) Kaplan–Meier survival curves showing the survival probability over time in the high and low invasion groups. (B, E) Boxplot of TIDE immunotherapy response status, showing the difference in the distribution of ANAPC13 + ductal or HECW2 + fib between immune responders and nonresponders. (C, F) Plot of correlation between ANAPC13 + ductal or HECW2 + fib and immune‐related markers.

We conducted differential analysis of ANAPC13‐positive ductal cells and HECW2‐positive fibroblasts to identify their respective marker genes. Based on these markers, RNA‐seq data were scored using ssGSEA. Patients were then classified into high‐ and low‐expression groups based on the median score for survival analysis. The results revealed that patients with low infiltration of ANAPC13‐positive ductal cell markers and HECW2‐positive fibroblast markers had a significantly better prognosis. This indicates a strong correlation between low ANAPC13 expression in ductal cells, low HECW2 expression in fibroblasts, and favorable tumor prognosis (Figure 6A,D).

Immunotherapy analysis further showed that patients with lower infiltration of ANAPC13‐positive ductal cell markers exhibited a more favorable response to immunotherapy (Figure 6B,E). This finding reinforces the relationship between reduced ANAPC13 infiltration in ductal cells and improved prognosis. Additionally, correlation maps illustrating the expression of ANAPC13‐positive ductal cells and HECW2‐positive fibroblasts with other immune cell populations and their respective functions were generated (Figure 6C,F). These maps revealed how interactions between different cell types in the TME influence tumor immune responses.

### 3.8. Screening of Common Differential Genes and Potential Role of LAMA3 in TME

**Figure 7:**
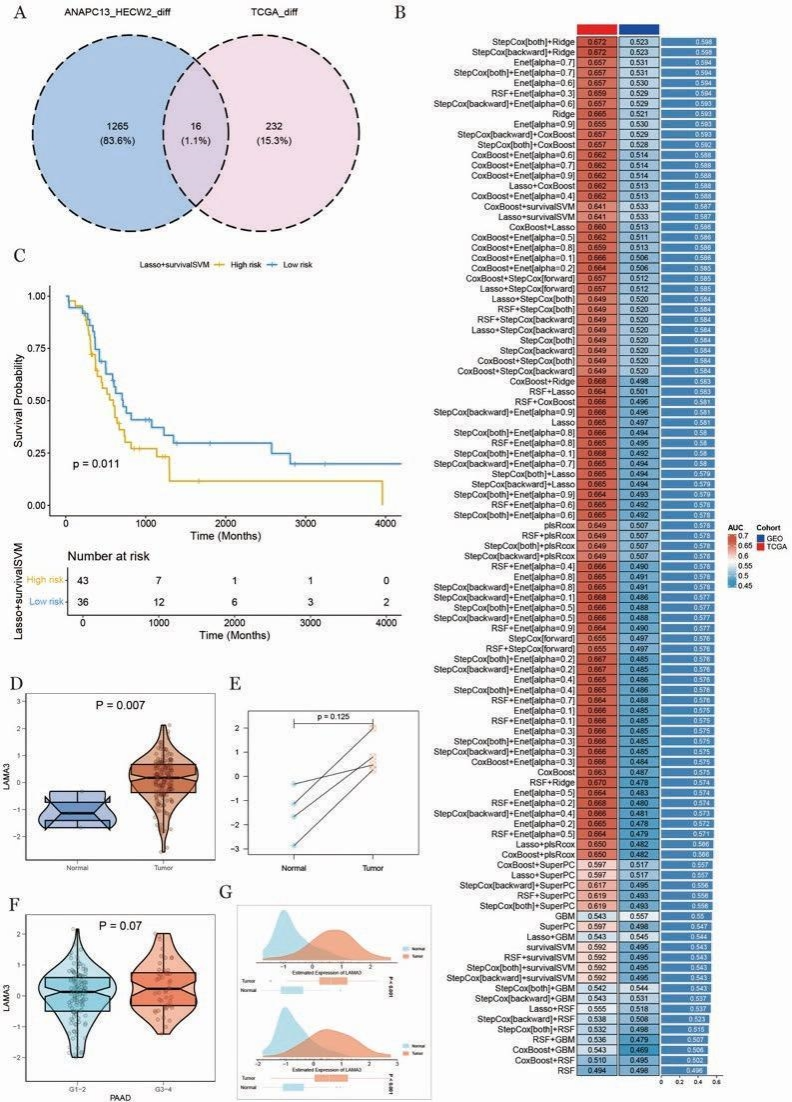
Machine learning of differential genes and differential analysis of LAMA3. (A) Intersection of differential genes in Venn diagram, (B) machine learning of common differential genes, (C) Lasso + survivalSVM model KM survival curve, and (D) LAMA3 expression difference between tumor and normal tissues in TCGA data, the upper and lower ends of the box indicate the quartile range of values. The line in the box represents the median (E) paired differential analysis of LAMA3 expression in tumor and normal tissues in TCGA data. (F) Differential expression of LAMA3 in high and low tumor grades in TCGA data. (G) Comparison of LAMA3 expression levels in normal and tumor tissues in the two GEO datasets.

We integrated differential gene data from ANAPC13‐negative and positive cells, as well as from HECW2‐negative and positive cells, and then intersected these with the differential genes obtained from TCGA data. This process identified 16 common differential genes, which were presented in a Venn diagram (Figure 7A). To further validate the clinical relevance of these genes, we performed machine learning analysis using TCGA data as the training set and GSE data as the validation set. A heat map was created to visualize the performance of different machine learning models on both datasets (Figure 7B). Based on the evaluation, the model with the highest performance on the validation dataset, namely lasso regression combined with survival support vector machine (Lasso + survivalSVM), was selected. Survival analysis was conducted by plotting KM survival curves for the high‐risk and low‐risk groups identified by the model. The results revealed that the low‐risk group exhibited a significantly better prognosis (Figure 7C).

From the machine learning results, six key genes associated with the model were identified. Among them, LAMA3, a gene more closely linked to ubiquitination, was further examined. Differential expression analysis and paired difference analysis were performed to assess the expression of LAMA3, with the results visualized in boxplots. It was found that LAMA3 expression was significantly higher in tumor tissues compared with adjacent normal tissues (Figure 7D,E). Furthermore, in the comparison between high and low grades, LAMA3 expression was elevated in higher‐grade tumors (Figure 7F), reinforcing its potential role in tumor progression.

To increase confidence in these findings, we repeated the differential expression analysis using two additional GEO datasets (GSE28735 and GSE62452). The results confirmed significantly higher LAMA3 expression in tumor tissues (Figure 7G), validating our earlier observations.

### 3.9. Screening of Common Differential Genes and LAMA3 in TME

**Figure 8:**
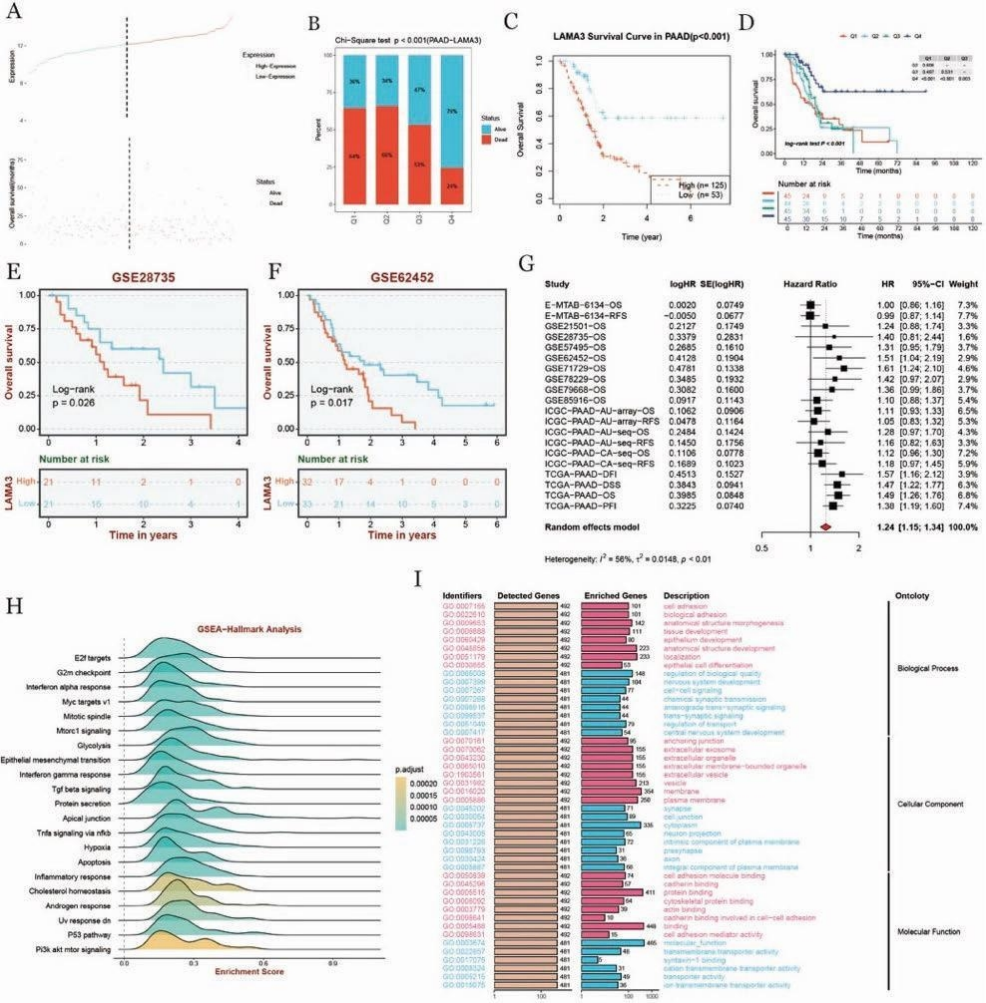
LAMA3 expression and survival analysis and functional enrichment results in pancreatic cancer patients. (A) Plot of LAMA3 expression and survival status, each scatter represents a patient, blue represents patient survival, red represents patient death. (B) Chi‐square test was performed on the number of survival and death samples with different LAMA3 expression levels. The *x*‐axis represents patients with different LAMA3 expression levels, and Q1 represents the highest 25% of samples. Q4 represents the lowest 25% of samples, the *y*‐axis represents the ratio of dead to alive in groups with different expression levels, blue represents survival, red represents the KM survival curve of death. (C) LAMA3 in pancreatic cancer TCGA data. (D) KM survival curve of LAMA3 in four survival periods (OS, DSS, PFI, and DFI) in pancreatic cancer TCGA data. (E, F) KM survival curve (G) of LAMA3 in GSE28735 and GSE62452 is the majority meta‐analysis of data set univariate Cox survival analysis, (H) hallmark gene set GSEA pathway enrichment analysis, and (I) GO functional enrichment analysis.

To investigate the association between LAMA3 gene expression and patient survival status, we visualized the relationship between LAMA3 expression levels and survival data in the form of a survival curve. The results demonstrated that patients with higher LAMA3 expression had a significantly greater number of deaths and shorter OS (Figure 8A). Further statistical analysis, specifically a chi‐square test, was conducted on the distribution of survival and death events based on LAMA3 expression levels, presented as a bar graph. The highest 25% of patients with elevated LAMA3 expression showed the highest incidence of death, suggesting a correlation between high LAMA3 expression and poor prognosis (Figure 8B).

KM survival analysis was performed using TCGA data for patients stratified by LAMA3 expression levels. The results revealed that patients with low LAMA3 expression had significantly longer survival times compared with those with high LAMA3 expression (Figure 8C). Similar trends were observed in the GSE28735 and GSE62452 datasets, further validating these findings (Figure 8E,F). A comparative analysis showed that the 25% of patients with the highest LAMA3 expression had markedly shorter survival compared with the 25% of patients with the lowest LAMA3 expression (Figure 8D).

To evaluate the prognostic value of LAMA3 in pancreatic cancer, survival analysis of multiple cohorts was performed, and survival results of different datasets were integrated by random effects model. The HR values of each cohort were mostly clustered above 1.0, indicating that LAMA3 was associated with cross‐over survival prognosis in most cohorts (Figure 8G). In addition, we also performed pathway enrichment analysis by GESA‐hallmark analysis, and the results showed that the pathways with high enrichment scores included E2F targets, G2m checkpoint, Interferon alpha response, glycolysis, and so on. It suggests that they may play an important role in tumorigenesis and development [38–40]. In addition, Epithelial mesenchymal transition, interferon gamma response, Tgf beta signaling and other pathways also showed significant enrichment, further indicating their potential functions in the tumor environment (Figure 8H). In order to further understand the biological functions of genes, GO enrichment analysis was performed to reveal the important roles of genes in key biological processes such as cell structure, signaling transduction and adhesion in the form of bar graphs (Figure 8I).

### 3.10. Genomic Alteration Landscape of LAMA3 in PDAC

**Figure 9:**
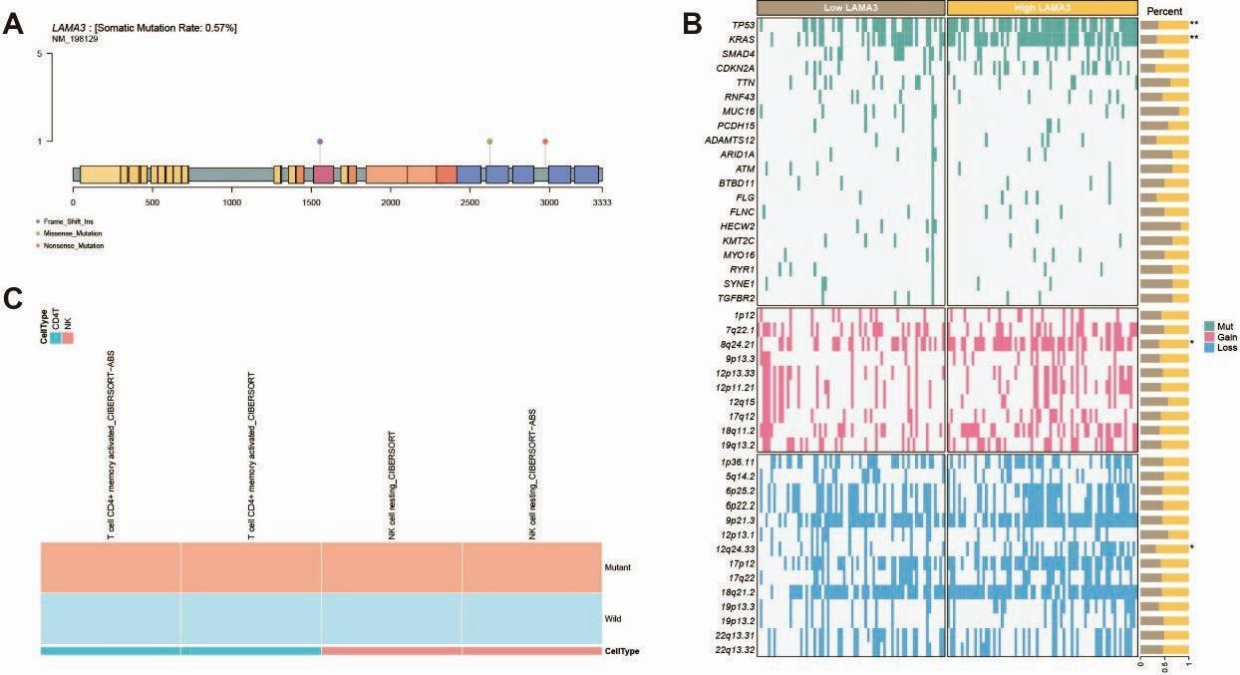
Genomic alteration landscape and immune microenvironment association of LAMA3 in pancreatic ductal adenocarcinoma. (A) Lollipop plot showing the distribution and types of somatic mutations in LAMA3 across PDAC samples from the TCGA cohort. Different colors indicate distinct mutation types, including missense, nonsense, and frameshift mutations. (B) Genomic alteration profiles of PDAC samples stratified by LAMA3 expression levels. The upper panel shows mutation patterns of common driver genes, whereas the middle and lower panels display copy number gain and loss events across genomic regions. Samples were grouped into LAMA3‐low and LAMA3‐high expression cohorts. (C) Comparison of immune cell infiltration between LAMA3 mutant and wild‐type samples based on TIMER2.0 immune estimation data from TCGA. Immune cell types showing significant differences (*p* < 0.001, Wilcoxon test) are presented in the heat map.

To further characterize the genomic features of LAMA3 in PDAC, we first analyzed its somatic mutation landscape in the TCGA cohort. The results showed that LAMA3 exhibited a relatively low mutation frequency, and the detected alterations were distributed across multiple regions of the protein sequence, including missense mutation, nonsense mutation, and frameshift insertion (Figure 9A). These findings provided an overview of the mutational pattern of LAMA3 in PDAC. We next compared genomic alteration profiles between the LAMA3‐high and LAMA3‐low expression groups. Oncoprint analysis showed that common driver gene mutations, including TP53, KRAS, SMAD4, and CDKN2A, were present in both groups, whereas differences in the frequencies of several genomic alteration events were also observed between the two groups (Figure 9B). In addition, copy number gain and loss events across multiple chromosomal regions were visualized, further outlining the genomic alteration landscape associated with different LAMA3 expression states. To preliminarily assess the relationship between LAMA3 alteration status and the TME, we further compared immune cell infiltration between LAMA3 mutant and wild‐type samples using immune estimation results from the TIMER2.0 platform. Significant differences were identified in several immune cell populations by multiple algorithms, and the significantly changed cell types were displayed in a heat map (Figure 9C). These results suggested that LAMA3 was associated with distinct genomic alteration patterns and immune infiltration features in PDAC.

### 3.11. LAMA3 Knockdown Suppresses Malignant Phenotypes of Pancreatic Cancer Cells

**Figure 10:**
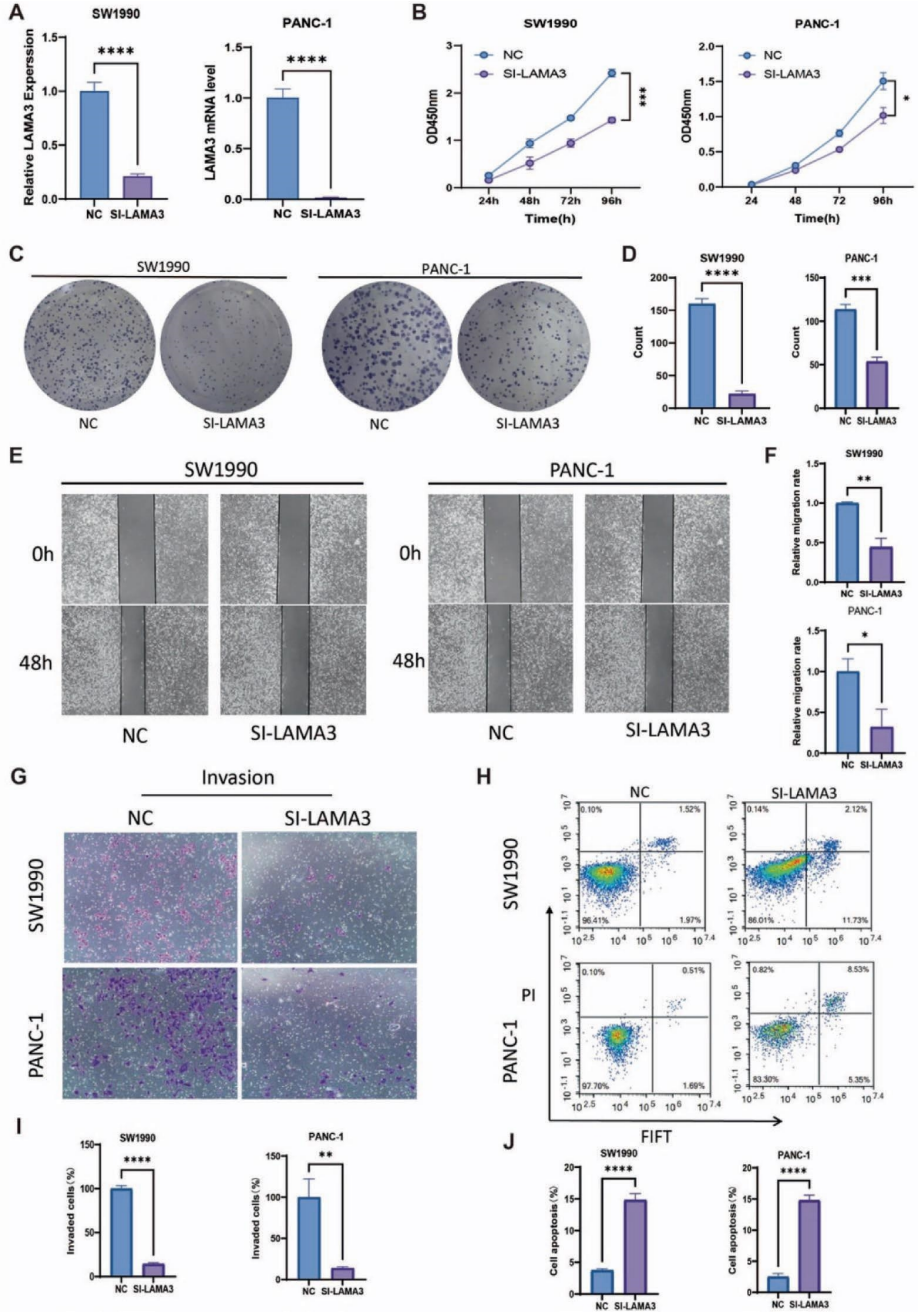
Knockdown of LAMA3 suppresses malignant biological behaviors in pancreatic cancer cells. (A) qRT‐PCR analysis confirmed that LAMA3 mRNA expression was significantly reduced in SW1990 and PANC‐1 cells after transfection with siRNA‐LAMA3 (SI‐LAMA3), compared with the negative control (NC). (B) CCK‐8 assay demonstrated that knockdown of LAMA3 significantly inhibited the proliferation of pancreatic cancer cells over time. (C) Colony formation assay showed a marked decrease in the number of colonies formed by cells transfected with SI‐LAMA3, indicating suppressed colony formation ability. (D) Quantitative analysis of colony numbers further confirmed that LAMA3 knockdown significantly reduced the colony‐forming capacity of SW1990 and PANC‐1 cells. (E) Wound healing assay revealed that knockdown of LAMA3 impaired the migration ability of pancreatic cancer cells, as evidenced by slower wound closure. (F) Quantification of wound closure rates further demonstrated that LAMA3 knockdown significantly reduced cell migration capacity. (G) Transwell invasion assay indicated that knockdown of LAMA3 significantly decreased the number of invasive cells, reflecting impaired invasion ability. (H) Flow cytometry analysis showed a significant increase in the apoptosis rate of pancreatic cancer cells after LAMA3 knockdown. (I) Quantification of invasive cell numbers in the Transwell invasion assay confirmed that knockdown of LAMA3 suppressed the invasion potential of SW1990 and PANC‐1 cells. (J) Statistical analysis of apoptotic cell percentages indicated that LAMA3 knockdown markedly promoted apoptosis in SW1990 and PANC‐1 cells.  ^∗^p < 0.05,  ^∗∗^p < 0.01,  ^∗∗∗^p < 0.001,  ^∗∗∗^p < 0.0001.

Knockdown of LAMA3 significantly inhibited the malignant biological behaviors of pancreatic cancer cells. qRT‐PCR confirmed that the expression of LAMA3 was successfully suppressed in SW1990 and PANC‐1 cells after transfection with siRNA‐LAMA3 (Figure 10A). Cell proliferation was significantly reduced in the siRNA‐LAMA3 group compared with the NC group, as demonstrated by the CCK‐8 assay (Figure 10B). Similarly, colony formation ability was markedly decreased in cells with LAMA3 knockdown, as shown by the colony formation assay (Figure 10C,D).

Furthermore, the wound healing assay revealed that LAMA3 knockdown substantially impaired the migratory capacity of pancreatic cancer cells, with reduced wound closure rates observed at 48 h (Figure 10E,F). In the Transwell invasion assay, the number of invaded cells was dramatically lower in the siRNA‐LAMA3 group than in the NC group, indicating that silencing LAMA3 significantly suppressed the invasive potential of SW1990 and PANC‐1 cells (Figure 10G,I).

In addition, flow cytometry analysis showed a significant increase in the apoptotic rate of pancreatic cancer cells following LAMA3 knockdown, suggesting that silencing LAMA3 promotes apoptosis in SW1990 and PANC‐1 cells (Figure 10H,J). Collectively, these results demonstrate that LAMA3 plays a critical role in promoting proliferation, migration, invasion, and survival of pancreatic cancer cells, and its knockdown effectively suppresses the malignant phenotypes associated with pancreatic cancer.

## 4. Discussion

PDAC is an extremely aggressive malignancy with a poor prognosis. Its high invasiveness and propensity for early metastasis often result in a late‐stage diagnosis, which significantly impacts treatment effectiveness and survival rates [1, 2].

Studies have demonstrated that cell interactions within the TME play a crucial role in cancer progression. These interactions are influenced by various factors, including cytokines, chemical signals, and the extracellular matrix [41, 42]. Ubiquitination, as a critical mechanism of intracellular signal transduction, plays a vital role in essential cellular processes such as cell proliferation, apoptosis, and protein homeostasis. Additionally, it significantly contributes to tumor initiation and progression. For instance, ubiquitination regulates tumor cell survival and proliferation by modulating the cell cycle, DNA repair mechanisms, and immune evasion [8, 9].

In this study, we comprehensively examined the biological significance of ubiquitination in PDAC by integrating scRNA‐seq, stRNA‐seq, and conventional RNA‐seq data. Through scoring the samples for ubiquitination levels, we observed that ubiquitination was significantly elevated in tumor tissues compared with adjacent normal tissues, highlighting its critical role in the TME. Statistical analysis revealed that ductal cells and fibroblasts exhibited notably higher ubiquitination scores in the tumor group. Combined with cell communication and CNV analysis, these two cell types were selected for further investigation. These cells not only contribute to tumor growth and metastasis in the microenvironment but may also facilitate drug resistance by promoting adaptive changes in tumor cells [43, 44]. Subsequent enrichment analysis revealed a significant upregulation of the ubiquitin‐mediated proteolysis pathway in both ductal cells and fibroblasts, a pathway known to be crucial for tumor cell survival and drug resistance. Based on these findings, we selected ANAPC13 and HECW2 for further investigation to explore their specific roles in tumor development. These two genes were chosen not only for their importance in the ubiquitination pathway but also for their potential influence on the biological behavior of tumor cells and their interactions within the TME [45].

By integrating single‐cell sequencing data with spatial transcriptome data, we were able to illustrate not only the spatial distribution and cell‐to‐cell interactions of each cell type but also the temporal migration and spatial localization of ANAPC13‐associated ductal cells and HECW2‐associated fibroblasts at the tissue slice level [46].This localization analysis enhances our understanding of cell interactions within the TME and their impact on tumor progression, particularly in the processes of tumor cell migration and invasion [47]. Furthermore, the analysis of the prognostic and immunotherapy effects of ANAPC13 in ductal cells and HECW2 in fibroblasts revealed that low expression levels of both genes were significantly associated with a more favorable tumor prognosis and a better response to immunotherapy [48]. This suggests that these two cell types not only play a critical role in tumor progression but may also serve as potential new therapeutic targets.

Subsequently, we integrated TCGA expression data and survival information, focusing on LAMA3 through differential gene intersection, machine learning, and the construction of survival curve models. By analyzing the differential expression of LAMA3 in tumor versus normal tissues using both TCGA and GEO data, we observed a significant increase in LAMA3 expression in tumor tissues, consistent with its role in cell migration and invasion. Further investigation of the relationship between LAMA3 expression levels and patient survival revealed that low LAMA3 expression was strongly associated with a better prognosis. This finding not only validates the biological function of LAMA3 in PDAC but also highlights its potential as a promising biomarker, which could provide novel strategies for personalized therapy, particularly for targeting the tumor immune microenvironment.

Finally, we confirmed that LAMA3 plays a key role in promoting the proliferation, migration, invasion, and survival of pancreatic cancer cells in vitro, and its knockdown can effectively inhibit the malignant phenotype of pancreatic cancer cells.

In conclusion, our study provides new insights into the complex biology of pancreatic cancer, emphasizing the crucial role of ubiquitination in cancer progression and uncovers the potential function of the LAMA3 gene in PDAC. Future research should further investigate the specific mechanisms linking LAMA3 and ubiquitination, particularly in regulating cell‐signaling pathways, altering the TME, and influencing immune responses. This will offer new perspectives for early diagnosis and targeted therapies for pancreatic cancer.

Nevertheless, several limitations should be acknowledged. This study was primarily based on publicly available datasets with relatively limited sample sizes and lacked external validation cohorts. These factors may constrain the generalizability of our findings. Moreover, given the inherent heterogeneity of PDAC, further validation in large, independent, and multicenter cohorts is necessary. Despite these limitations, our integrative approach provides a valuable framework for understanding the role of ubiquitination in PDAC and highlights promising molecular targets for future investigation.

## 5. Conclusions

This study reveals that ubiquitination‐associated regulatory programs critically shape ductal–fibroblast crosstalk in PDAC, thereby influencing tumor progression, invasive behavior, and clinical outcomes. Aberrant ubiquitination activity within ductal cells and fibroblasts contributes to microenvironmental remodeling and may underlie heterogeneous responses to therapy. Notably, LAMA3‐associated programs reflect aggressive tumor states and stromal interaction, highlighting their potential value for risk stratification and therapeutic targeting. Collectively, these findings provide a mechanistic framework linking ubiquitination‐driven cell–cell interactions to disease progression and support their relevance as candidates for translational biomarker development and microenvironment‐oriented treatment strategies in pancreatic cancer.

## Author Contributions

Y.F., X.Z., L.J., S.Z., P.X., and X.Z. contributed to the conceptualization of the article. Data curation and formal analysis were carried out by Y.F., X.Z., L.J., S.Z., Y.G., and G.H. Visualization was undertaken by Y.F., L.J., Z.Z., and X.Z. The original draft was written by Y.F., X.Z., L.J., S.Z., Y.G., G.H., Z.Z., and Z.X. The review and editing process involved P.X. and X.Z. Y.F., X.Z., L.J., and S.Z. contributed equally to this work.

## Funding

This study was supported by Sichuan Medical Science and Technology Innovation Research Association (YCH‐KY‐YCZD2024‐298); Southwest Medical University 2024 innovative training program for college students (S202410632176)

## Conflicts of Interest

The authors declare no conflicts of interest.

## Data Availability

The datasets analyzed in this study can be found in the Gene Expression Omnibus (GEO): single‐cell sequencing data from dataset GSE212966 at https://www.ncbi.nlm.nih.gov/geo/query/acc.cgi?acc=GSE212966; single‐cell sequencing data and spatial transcriptome data in dataset GSE235315 at https://www.ncbi.nlm.nih.gov/geo/query/acc.cgi?acc=GSE235315. The ubiquitylated gene set was obtained from the GO database at https://amigo.geneontology.org/amigo/search/bioentity?q=ubiquitination; and extensive sequencing data for TCGA pancreatic cancer from the Xena database at https://xenabrowser.net/datapages/?cohort=GDC%2520TCGA%2520Pancreatic%2520Cancer%2520(PAAD)%26removeHub=https%253A%252F%2525http://2Fxena.treehouse.gi.ucsc.edu%253A443. In addition, all analytical codes, genomes, raw images involved in this study and raw data have been uploaded to https://jianguoyun.com and can be accessed at https://www.jianguoyun.com/p/DfvTt4gQ85jYCxjPtPIFIAA.
